# Supplementary material for: HPV/E7 induces chemotherapy‐mediated tumor suppression by ceramide‐dependent mitophagy
Source: EMBO Mol Med. 2017 Jun 12;9(8):1030–51. doi: 10.15252/emmm.201607088 (PMC5538428; doi:10.15252/emmm.201607088)
Supplement: Supplementary file 1 — Expanded View Figures PDF [file EMMM-9-1030-s001.pdf]

Expanded View Figures

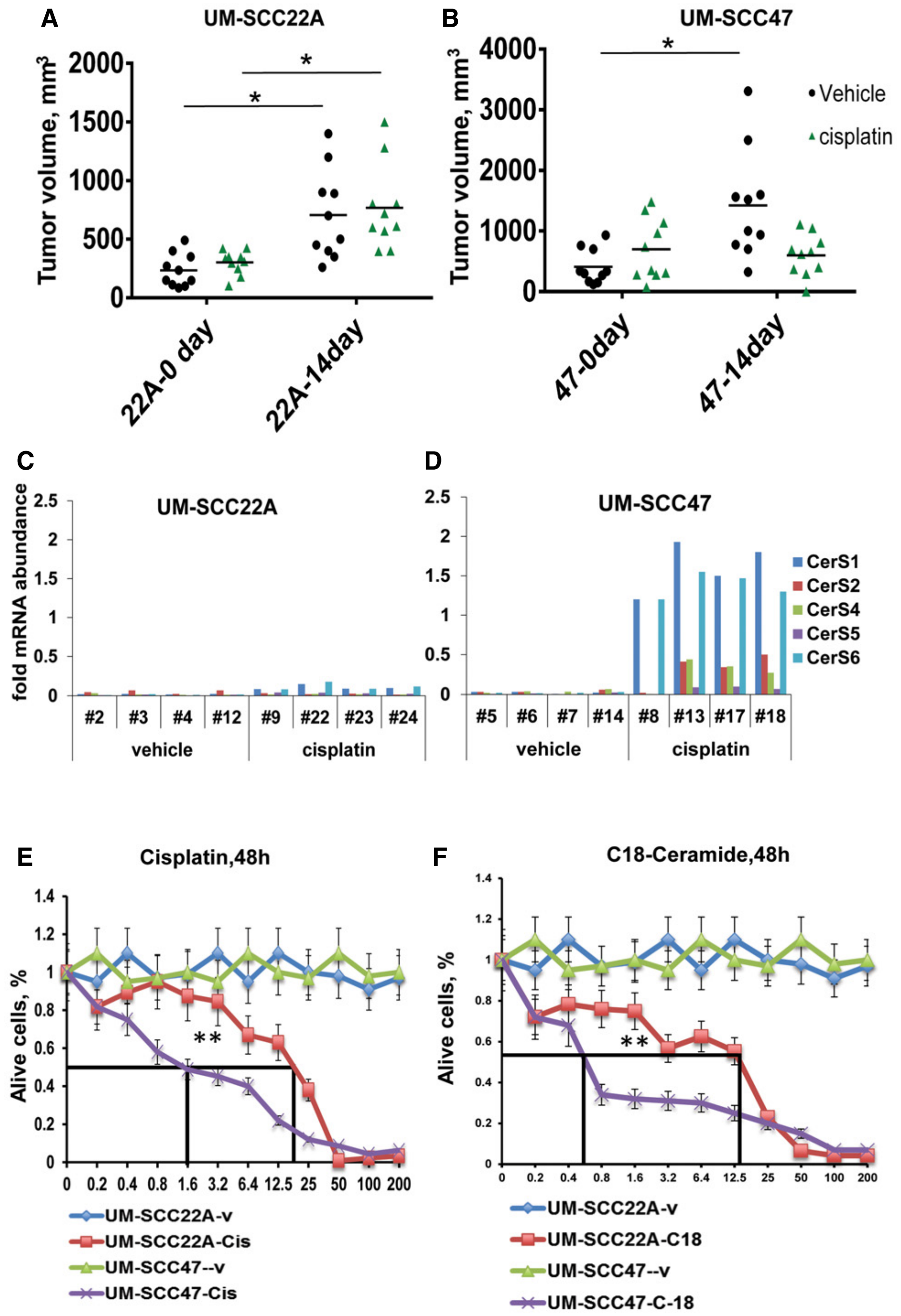

Figure EV1. HPV infection induces tumor suppression and cell death in response to cisplatin.

A–D Effects of cisplatin (3.5 mg/kg at 72-h intervals) on UM-SCC-22A (A) versus UM-SCC-47 (B)-derived xenograft tumors grown in the flanks of SCID mice were measured at days 0 and 14 (n = 5–8 mice/group, and $*P < 0.05$ by unpaired Student's t-test). After the measurement of tumor volumes, CerS1-6 mRNAs were measured in extracted tumor tissues from individual SCID mice treated with cisplatin at day 0 versus day 14 in UM-SCC-22A- (C) or UM-SCC-47 (D)-derived xenograft tumors. CerS1-6 mRNAs were normalized to 28S rRNA.

E, F Effects of cisplatin (E) and C_{18}-pyr-cer (F) on cell death (48 h) in UM-SCC-22A [HPV(−)] versus UM-SCC-47 [HPV(+)] cells were measured by trypan blue exclusion assay. Data are means ± SD from three independent experiments, analyzed by unpaired Student's t-test ($**P < 0.05$).

© 2017 The Authors

Figure EV2. Drp1 knockdown prevents mitophagy.

A Extended time course of experiment shown in Fig 2D. Effects of shRNA-mediated knockdown of Drp1 on mitophagy were measured using live cell imaging for co-localization of MTR/LTG in UM-SCC-1A cells transfected with vector (V) or HPV-E7 (E7 o/v) in the absence/presence of cisplatin (10 μM) for 15, 45, 60, and 120 min. Vehicle-treated cells were used as controls. Images represent at least three independent studies, and scale bar represents 100 μm.

B Ectopic expression of HPV-E7 was confirmed by qPCR (left panel), whereas Drp1 knockdown was confirmed by Western blotting (right panel) in cells presented in (A). Data represent three independent studies ± SD (n = 3).

C Representative graph obtained from Seahorse measurement of OCR in UM-SCC-47 cells grown in the absence/presence of C_{18}-pyr-cer (20 μM, 2 h) with appropriate inhibitors (as described in Materials and Methods) is shown. Data represent three independent studies ± SD (n = 3).

© 2017 The Authors

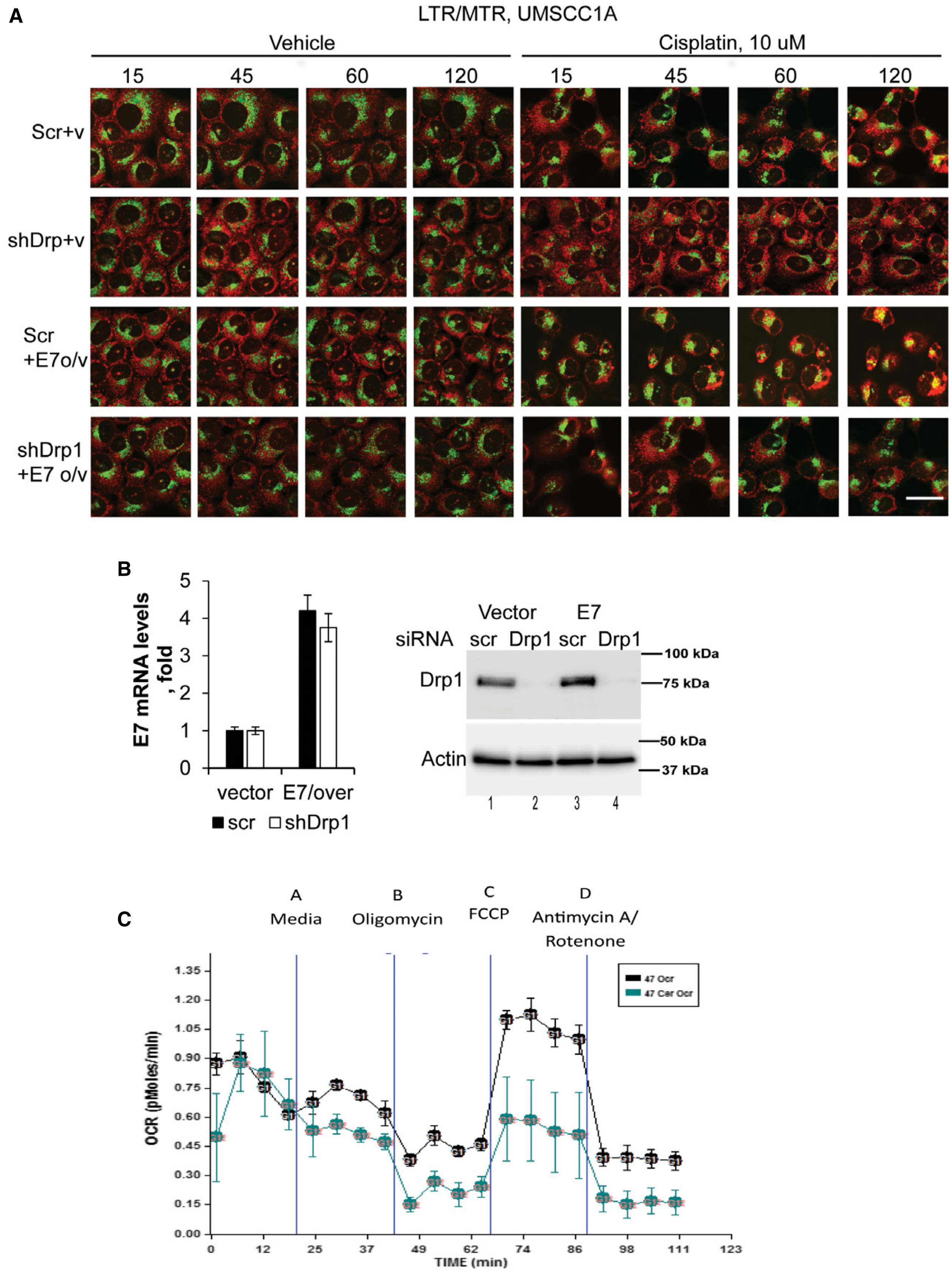
A
LTR/MTR, UMSCC1A
Vehicle
Cisplatin, 10 uM
15
45
60
120
Scr+v
shDrp+v
Scr +E7o/v
shDrp1 +E7 o/v
B
E7 mRNA levels , fold
vector
E7/over
scr
shDrp1
Vector
E7
siRNA
scr
Drp1
Drp1
Actin
100 kDa
75 kDa
50 kDa
37 kDa
C
A Media
B Oligomycin
C FCCP
D Antimycin A/ Rotenone
OCR (pMoles/min)
TIME (min)
47 Ocr
47 Cer Ocr

Figure EV2.

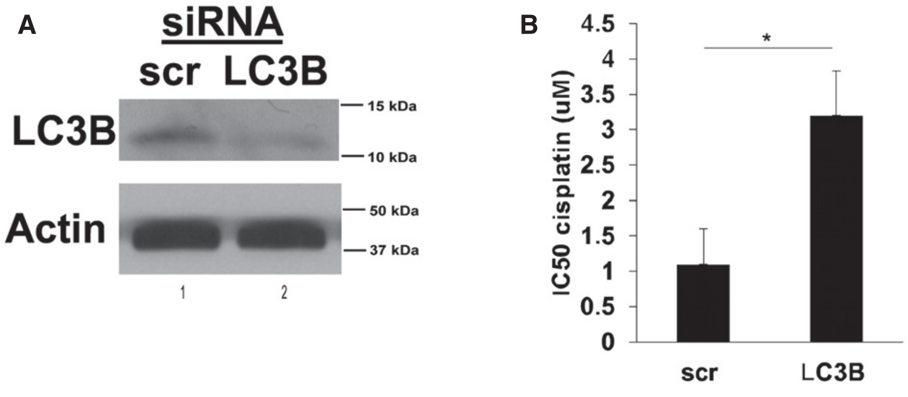

Figure EV3. Knockdown of autophagy inducer LC3 inhibits cisplatin-mediated cell death in HPV (+) HNSCC cells.

A, B siRNA-mediated knockdown of LC3B was confirmed using Western blotting with anti-LC3B antibody compared to Scr-siRNA-transfected controls (A). Actin was used as a loading control, and blots are representative of three independent experiments. Effects of siRNA-mediated knockdown of LC3B on cisplatin-mediated cell death were measured, and IC_{50} concentrations were calculated by MTT assays (B) compared to Scr-siRNA-transfected controls (48 h). Data are means ± SD from three independent experiments, analyzed by unpaired Student's *t*-test (*$P < 0.05$).

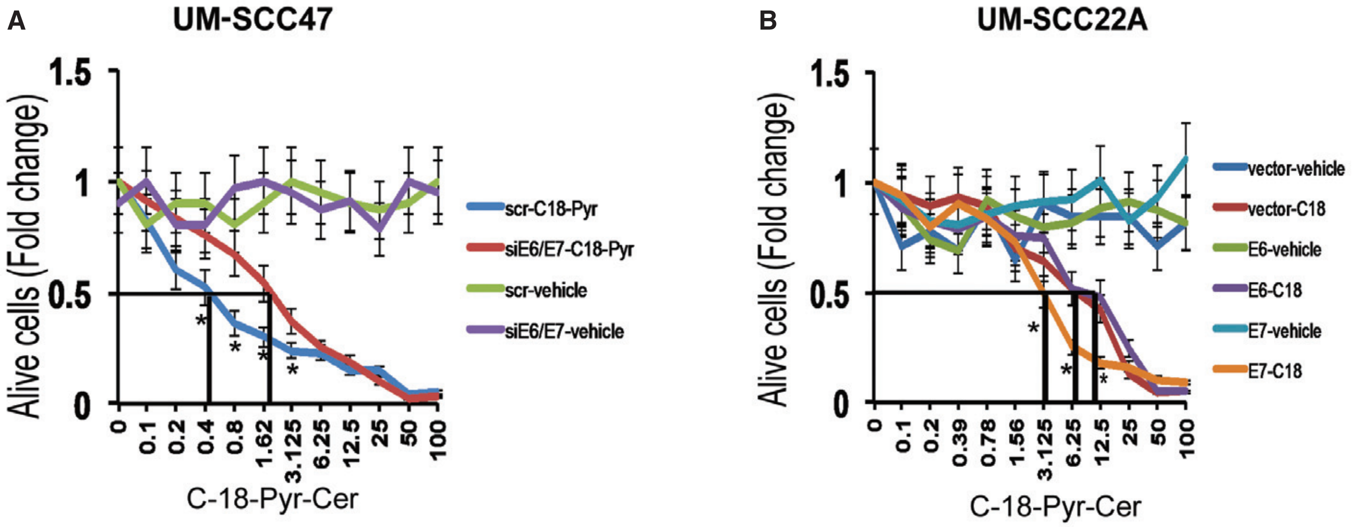

Figure EV4. HPV-E7 enhances mitochondrial ceramide-dependent lethal mitophagy.

A Effects of siRNA-mediated knockdown of HPVE6/E7 on UM-SCC-47 growth inhibition in response to C_{18}-pyr-cer (72 h) or vehicle (DMSO) were measured by MTT assay. Scr-siRNA-transfected cells were used as controls. Data are means ± SD from three independent experiments, analyzed by unpaired Student's *t*-test (*$P < 0.05$).

B Effects of ectopic expression of HPV-E6 versus HPV-E7 on HPV(−) UM-SCC-22A growth inhibition in response to C_{18}-pyr-cer (72 h) or vehicle (DMSO) were measured by MTT assay. Vector-transfected cells were used as controls. Data are means ± SD from three independent experiments, analyzed by unpaired Student's *t*-test (*$P < 0.05$).

 © 2017 The Authors

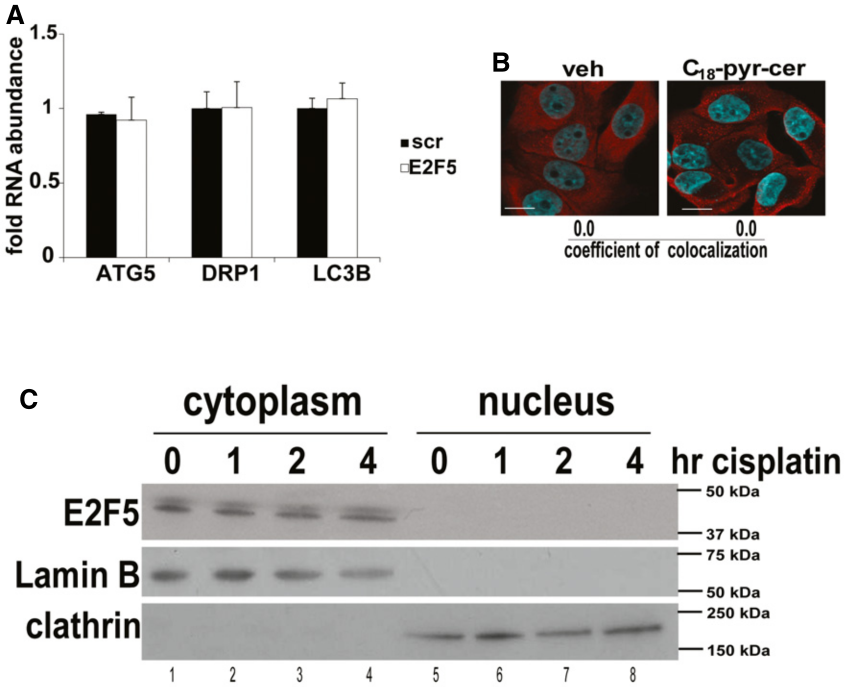

Figure EV5. Analysis of subcellular localization and function of E2F5 in HPV(+) HNSCC cells.

A Effects of shRNA-mediated knockdown on E2F5 on ATG5, Drp1, and LC3B mRNAs were measured using qRT–PCR in UM-SCC-47 cells compared to Scr-shRNA-transfected controls. Data are means ± SD from three independent experiments.

B Subcellular localization of E2F5 was assessed in the presence/absence of C_{18}-pyr-cer (20 μM, 1 h) by immunofluorescence using fixed confocal micrographs of UM-SCC47 cells stained with DAPI, anti-F-actin, and anti-E2F5 antibodies. Images represent at least three independent experiments. Scale bars represent 100 μm.

C Protein abundance of E2F5 in cytoplasm versus nucleus in the presence/absence of cisplatin (20 μM, for 0, 1, 2, and 4 h) was detected by Western blotting using cytoplasm- versus nuclei-enriched subcellular fractions of UM-SCC-47 cells using anti-E2F5 antibody. Anti-clathrin antibody was used to validate cytoplasmic fractions, whereas anti-lamin B antibody was used to validate nuclear fractions. Western blot images represent at least three independent experiments.

© 2017 The Authors
