# Supplementary figures and images for: HPV/E7 induces chemotherapy‐mediated tumor suppression by ceramide‐dependent mitophagy
Source: EMBO Mol Med. 2017 Jun 12;9(8):1030–51. doi: 10.15252/emmm.201607088 (PMC5538428; doi:10.15252/emmm.201607088)

Fig.1A

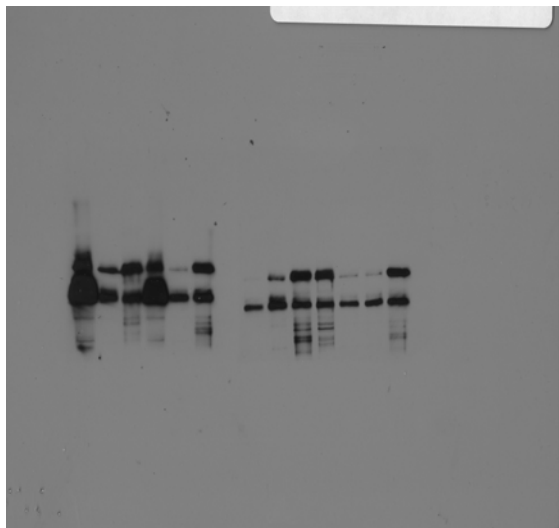

Fig.1b

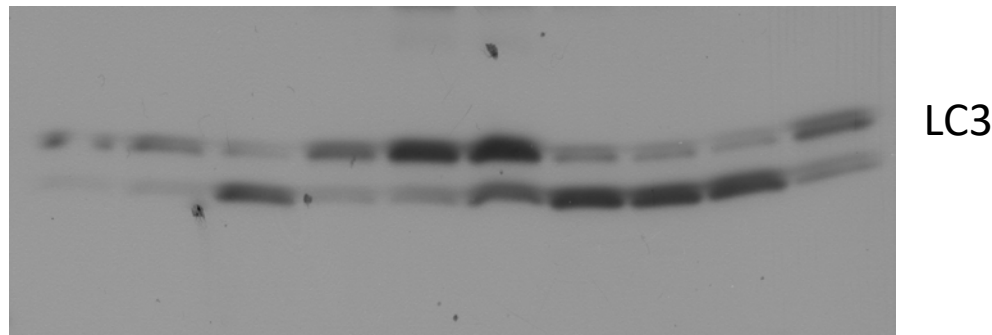

Fig.1f

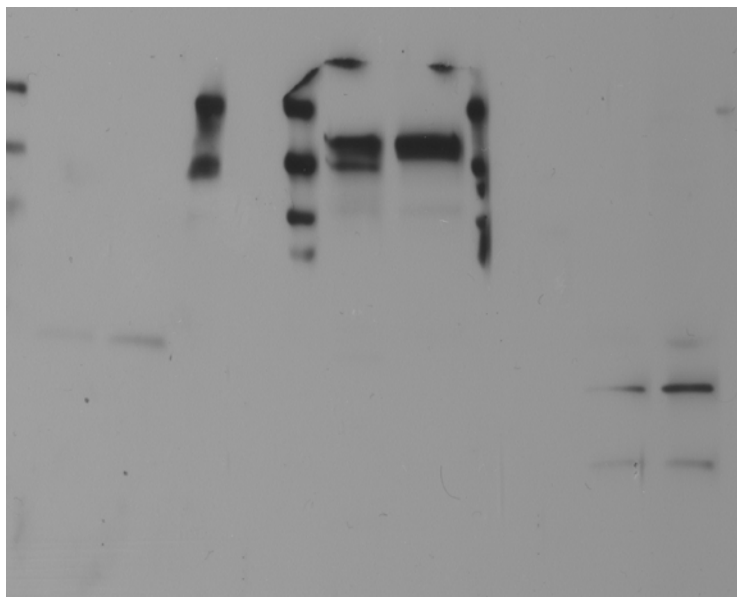

CerS1

Fig.1d, f

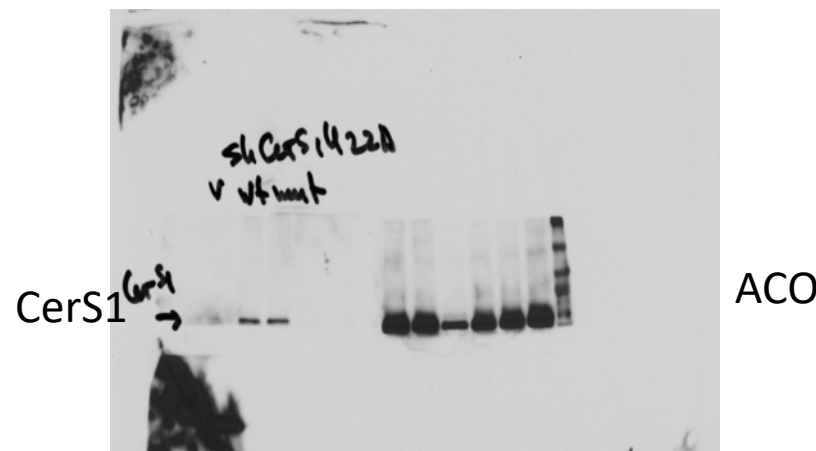

CerS1

ACO

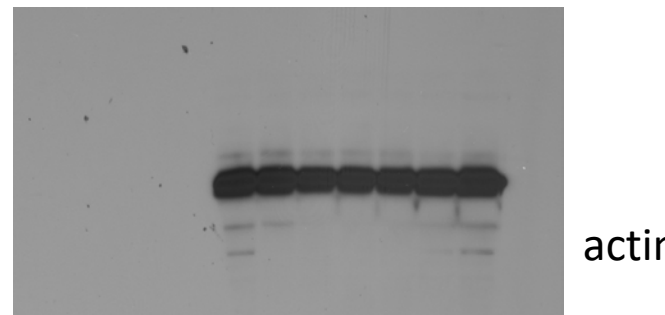

actin

Supplement: Supplementary file 3 — Source Data for Figure 1 [file EMMM-9-1030-s002.pdf]

**Fig. 2G**

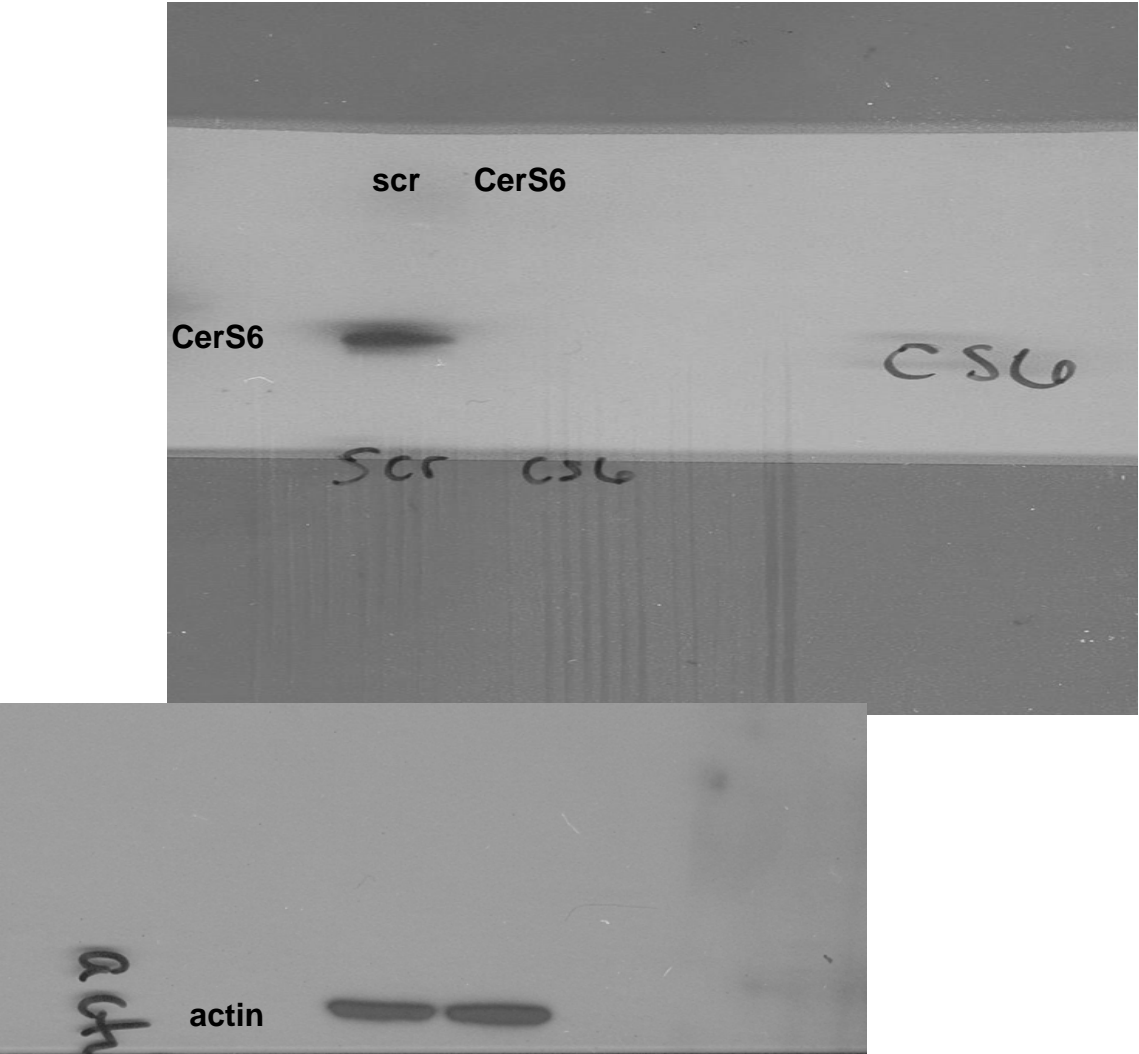

Supplement: Supplementary file 4 — Source Data for Figure 2 [file EMMM-9-1030-s003.pdf]

**Fig. 5A**

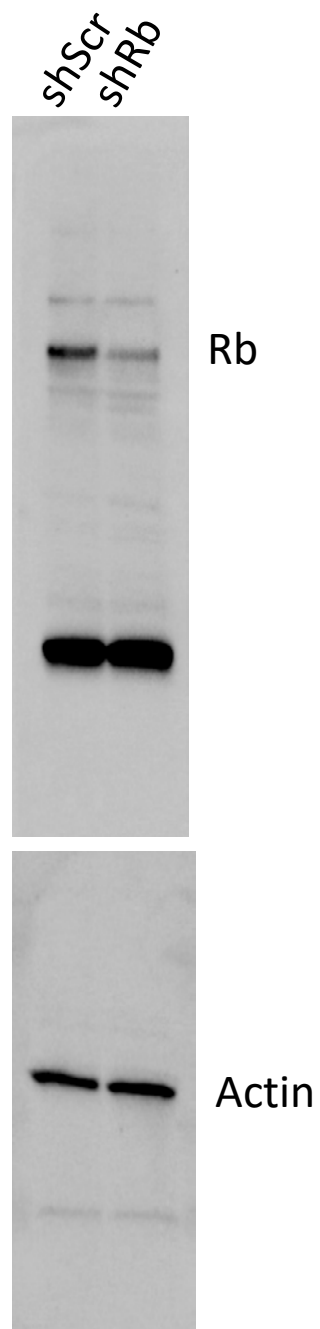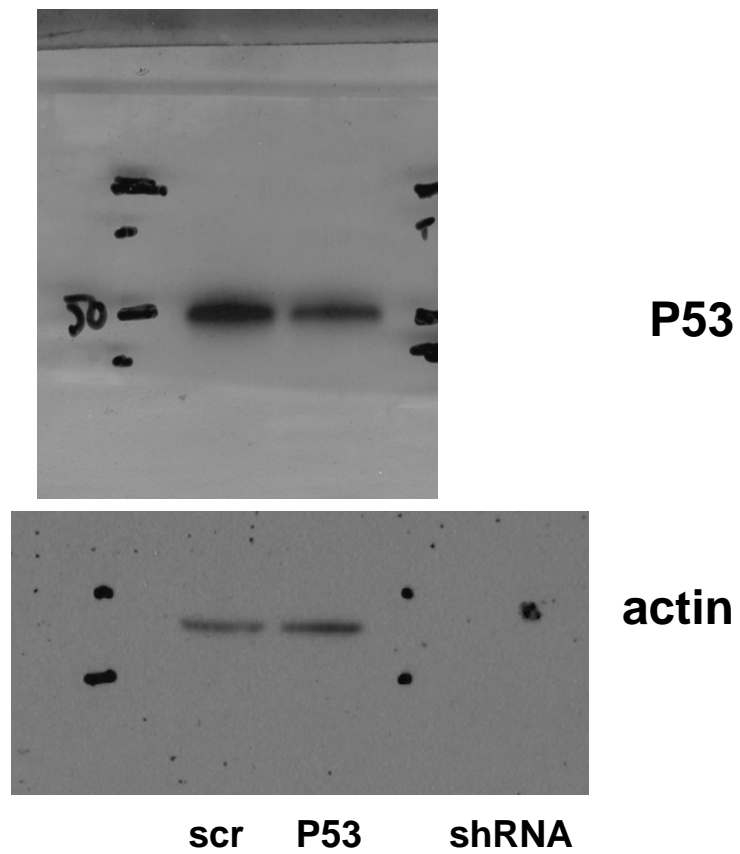

Supplement: Supplementary file 5 — Source Data for Figure 5 [file EMMM-9-1030-s004.pdf]

**Fig. 6D**

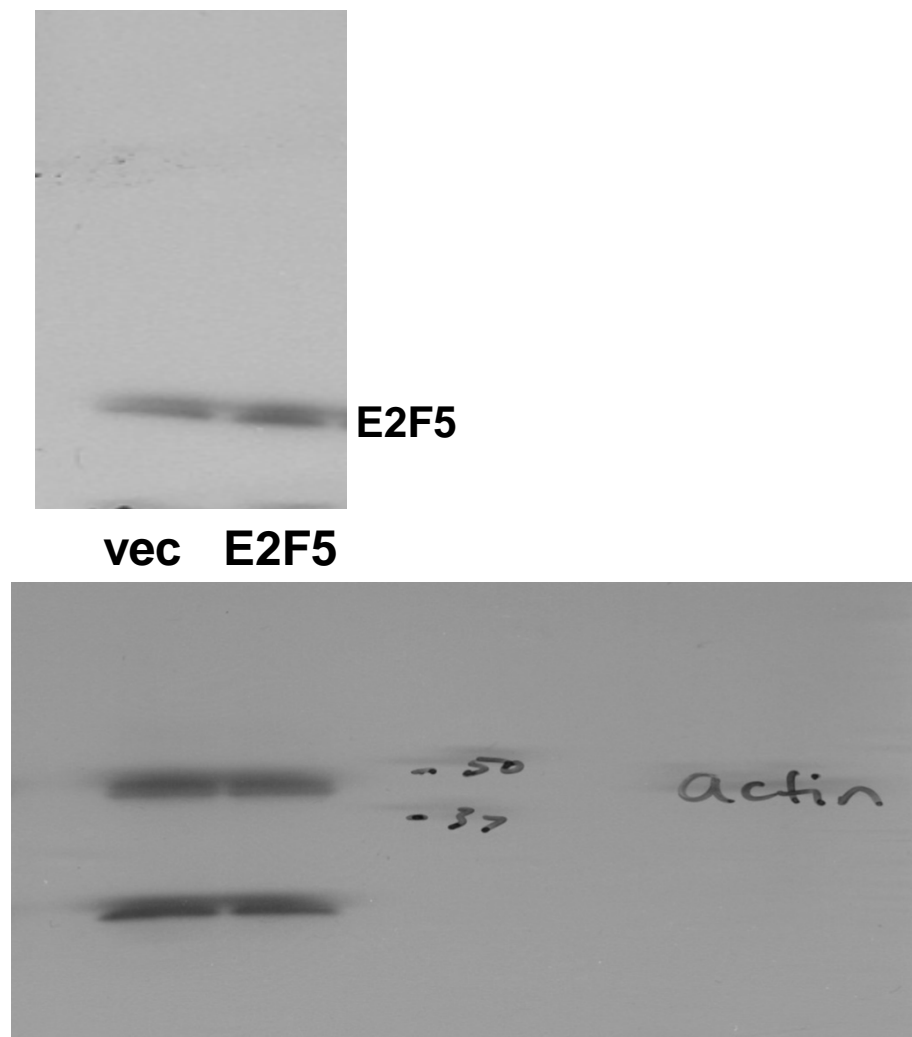

Supplement: Supplementary file 6 — Source Data for Figure 6 [file EMMM-9-1030-s005.pdf]

Fig. 7A

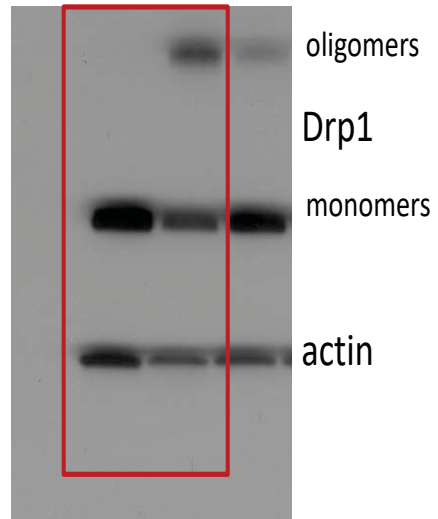

Fig. 7C

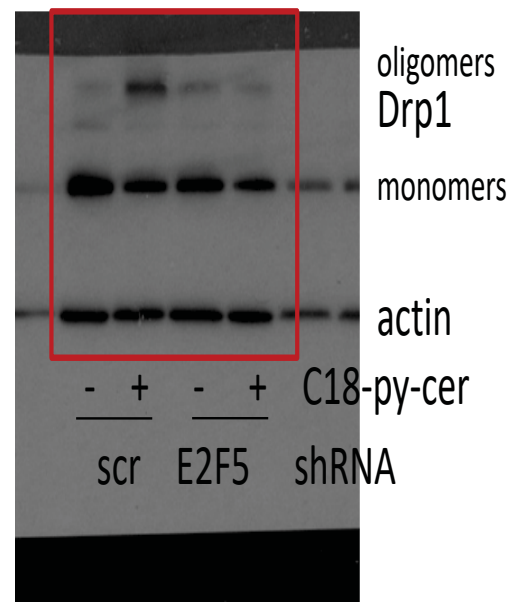

Supplement: Supplementary file 7 — Source Data for Figure 7 [file EMMM-9-1030-s006.pdf]

Fig.10G

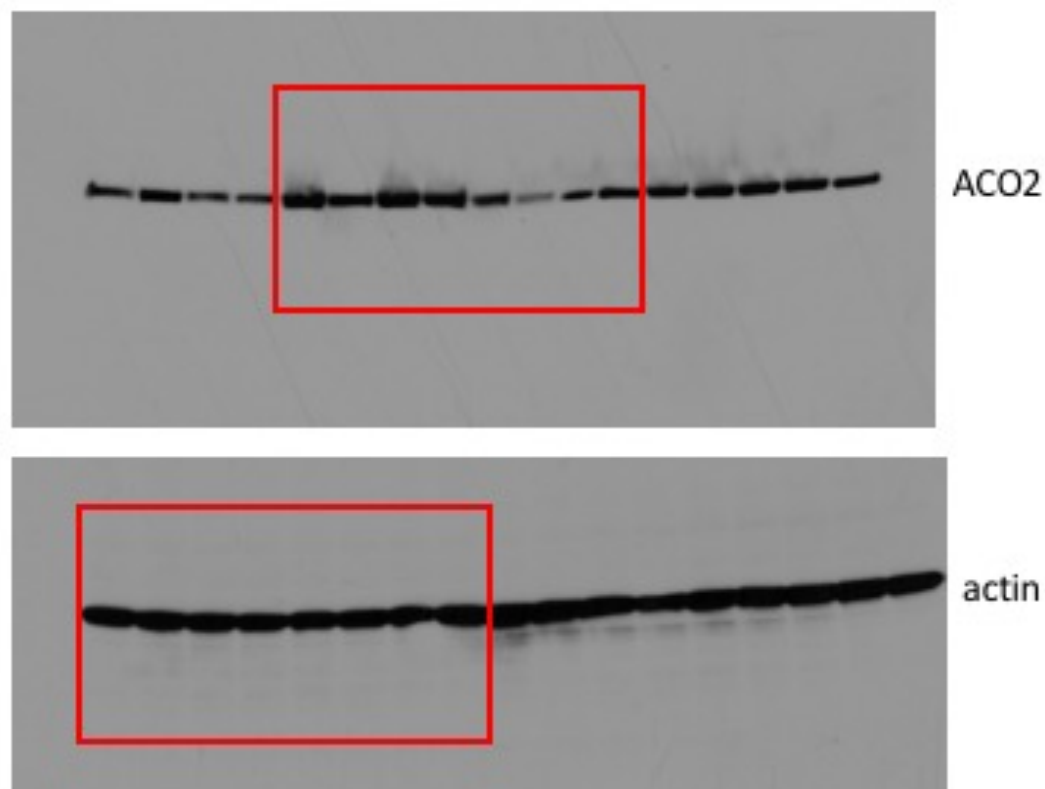

Fig.10k

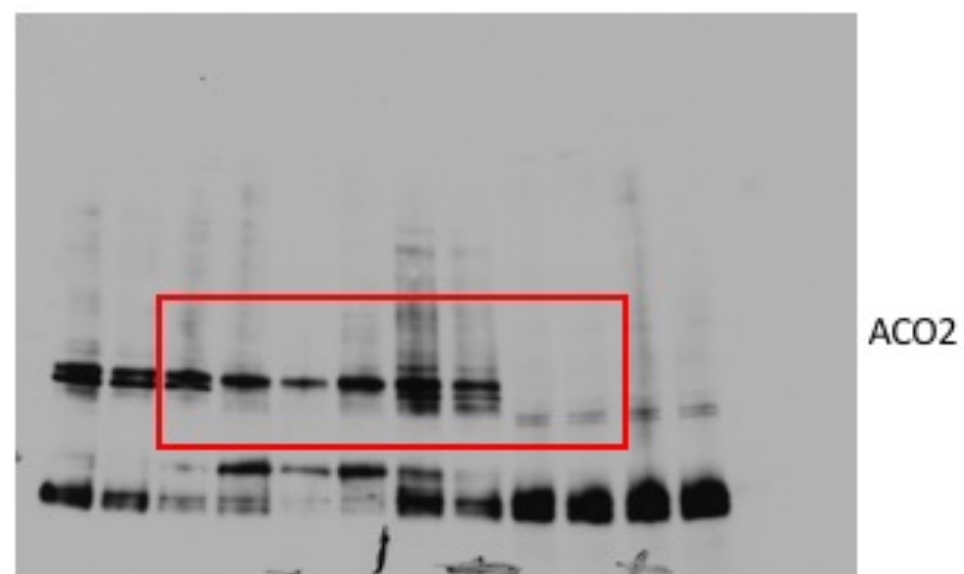

Supplement: Supplementary file 9 — Source Data for Figure 10 [file EMMM-9-1030-s008.pdf]
