# Supplementary material for: HPV/E7 induces chemotherapy‐mediated tumor suppression by ceramide‐dependent mitophagy
Source: EMBO Mol Med. 2017 Jun 12;9(8):1030–51. doi: 10.15252/emmm.201607088 (PMC5538428; doi:10.15252/emmm.201607088)
Supplement: Supplementary file 8 — Source Data for Figure 9 [file EMMM-9-1030-s007.pdf]

Fig. 9C-upper right panel

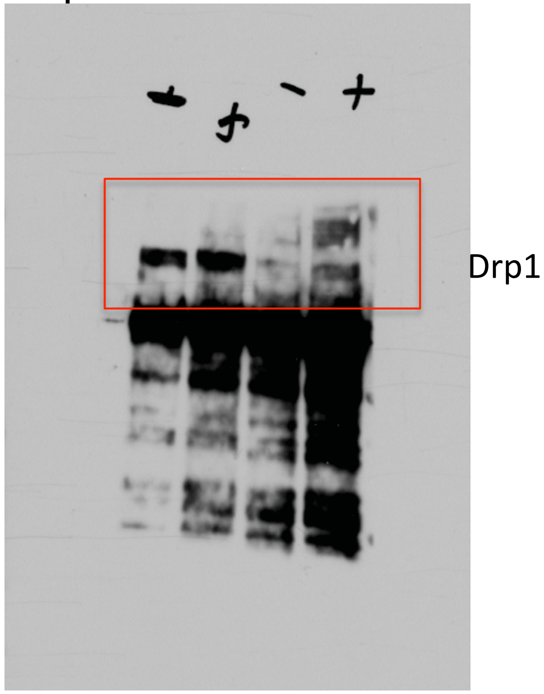

Drp1

Fig. 9D-upper right panel

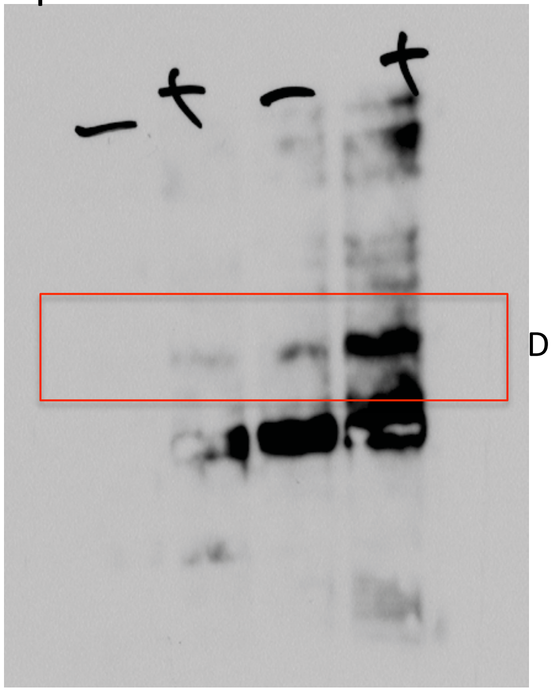

Drp1

Fig. 9D-upper left panel

Drp1

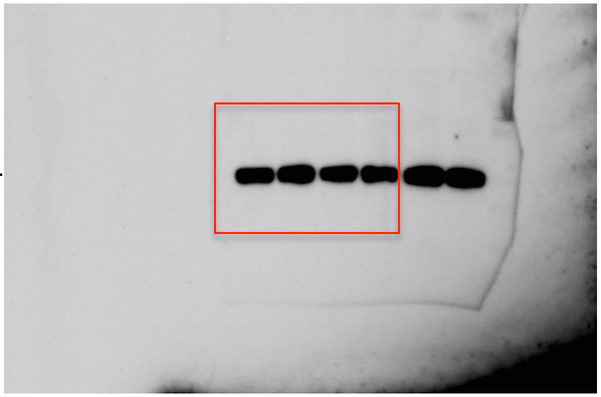

Fig. 9D-lower left panel

Drp1

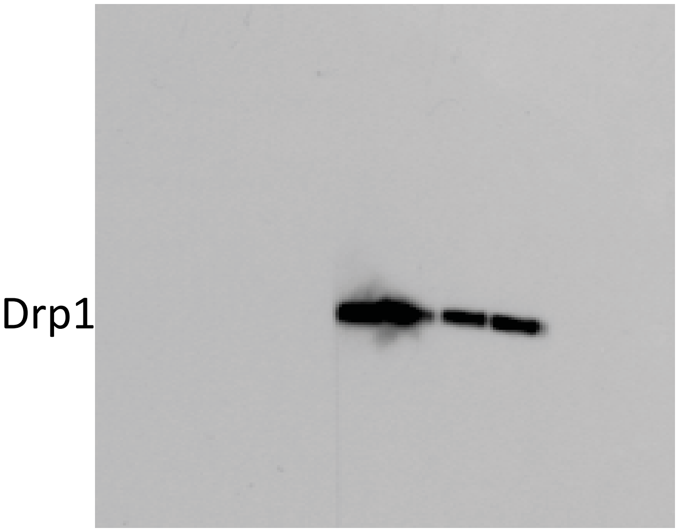

Fig. 9E

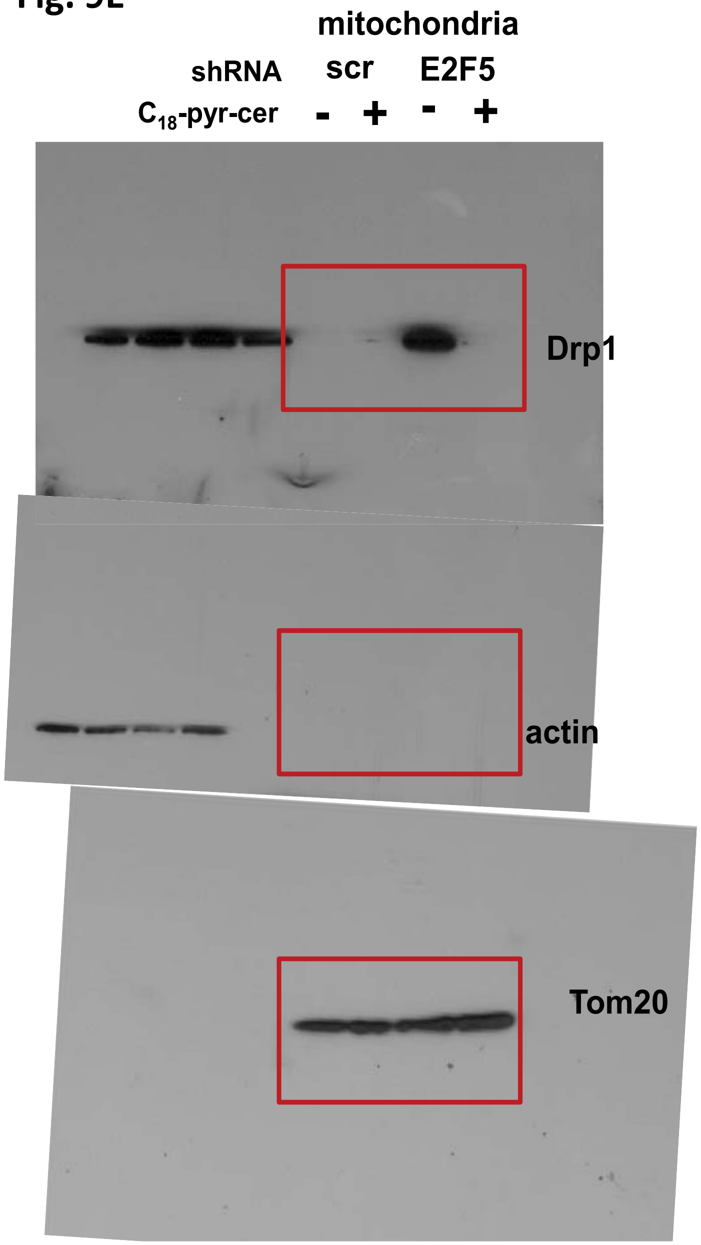

right panel

9F

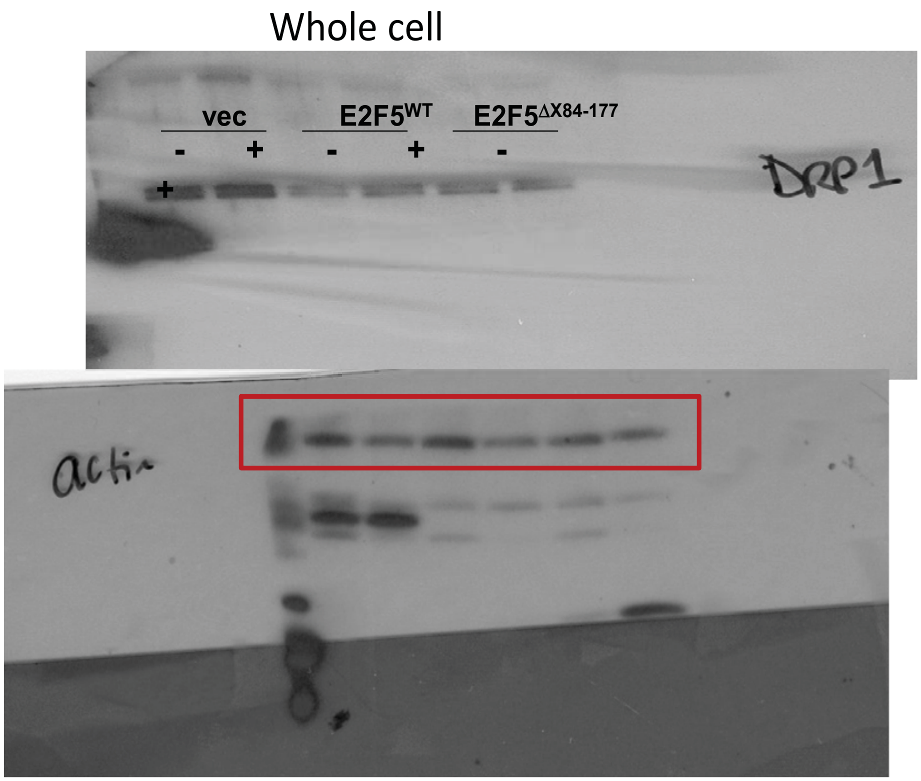

left panel
